# Revelations by Electricity

**Published:** 1885-12

**Authors:** F. T. Paine

**Affiliations:** Comanche, Texas


### REVELATIONS BY ELECTRICITY.

*By F. T. Paine, M. D., Comanche, Texas.*

(For Daniel's Texas Medical Journal.)

THE first of the cases in this series presented itself at my Clinic February 26, 1885. A girl thirteen years of age, Miss S. P., who, according to her mother's account, had menstruated regularly from March, 1883, to March, 1884, when the catamenia were suddenly suppressed from exposure. She had, at ten years of age, had the prodromes of approaching puberty or menstruation, for which I had prescribed. After the suppression in March, 1884, until September following, there was little or no trouble.

February 10, 1885, I was called to see her in a violent attack of spasmodic hysterical *dyspnœa;* very alarming to family and friends. She was soon relieved temporarily, but the attacks with short intervals, continued to recur; and I advised electricity. Her first seance was on the 26th of February, 1885. The feet and ankles failed to respond to a strong Faradic current. Over the fundus uteri was a small space, hyperæsthetic to the touch, but anæsthetic to electricity, and the hyperæsthesia was soon removed by the electric current. A spot, the size of a silver dollar over each ovary, was also found to be anæsthetic. Finding no pudendal development indicating puberty, I had her bosom exposed, and found no development of mammary glands whatever. A pale-blue circular mark, like that of a pencil, indicated the places of the future nipples.

On using the hand electrode, I found those spots entirely anæsthetic. As powerful a current as I could possibly bear to pass through the finger, was unfelt by the patient.

On a subsequent occasion I found the pudendum, vulva, labia minora and clitoris entirely insensible to electricity.

On the 3rd of March I directed the current to the mammary spots for five minutes each, and on the 9th of March, only six days afterwards, I found mammary enlargement, with nipples a quarter of an inch in length. The glands were half as large as a hen-egg, and from this date my patient continued to improve, until her mother reports her in perfect health.

After a few seances the response to the Faradic current was perfect. The menstrual action reported as having been regular, prior to this treatment, was pathological rather than physological, and was precocious.

The second case, Miss M. P., aged 22, had menstrated since her thirteenth year. At this date, February 26, 1885, memstruated imperfectly every 21 to 24 days, secretion dark—tarry; could not be washed from her clothes. An invalid for four years, under treatment of four physicians.

I found her feet and legs purple in spots, - her ankles swollen and anæsthetic to the electric current. Breasts resembled empty leathern sacks, nipples could have been made to meet over the back,[?]and were entirely *anæsthetic*, the whole breasts entirely insensible to any power of electricity I could bring to bear for several seances. On further investigations I found the lower half of the patient from lumbos-acral junction to feet, (except an inch or two on the inside of the upper thigh), the external and internal genitalia, entirely insensible to the Faradic current.

Never having had or heard of such cases, I was, of course, very much surprised, and began to look out for more of the same sort, and have not been long in finding many. In fact I have found every woman, who has come under my observation, who has been found to be the subject of sexual troubles, the subject also of electrical anæsthesia, so that I have established for myself a certain set of tests, or a system of diagnosis.

For instance, when a female applies at my Clinic for treatment, I ask permission to touch the ankle with an electrode. If the ankle responds to the current, I now go no further in that line; but if there is anæsthesia, then I apply an electrode to the breast, and if this gives no response, I am sure of finding anæsthesia of the ovarian regions—and have never yet been disappointed.

In the past nine months I have examined and treated quite a number of females, and have invariably found the above electrical symptoms, accompanied by a state of nervous exhaustion, that might be appropriately diagnosed *neurasthenia.*

Most of such patients suffer with some form of dyspepsia, and generally the longer standing the case, the more are the dyspeptic symptoms. Some have had strange hallucinations, some are demented to some extent. All, all, so far as I could ascertain from themselves or husbands, were, to a greater or less extent, devoid of sexual feeling. In two cases dysparreunia existed; one had separated from her husband, and one said she could not tolerate the marital act now; that her husband was kind and considerate and seldom taxed her.

I found this state to exist in delayed puberty, in dysmenorrhœa, in menorrhagia and in leucorrhœa, and in all abberations of the ovarian and uterine functions. So that when I find a female anæsthetic in her lower limbs and breasts, or in the ovarian region, I confidently expect to find some uterine or ovarian trouble coexisting.

It would worry my readers to read my cases. Two cases were more or less anæsthetic to the top of the head; both suffered from insomnia; both were mentally deficient or nearly lunatics. One bears a strong current on the anterior fontanelle which would nearly kill a healthy woman.

One could not feel a steel electrode introduced up the vagina to the os uteri, which the same woman would have thought red hot if put in her hand or applied to any other part. One had a steel electrode in the rectum for three seances of an hour each, before she could feel it at all—with all the power of a Kidder No. 5 tip battery.

These will show something of the insensiblity of so many of our females from whom we hear so much complaint, and of whose sufferings we can form no adequate conception, because we can see no local cause and no inflammatory action.

On the other hand I have found scarcely a man in whose lower limbs or breasts there was a loss of feeling to the current, and wherever I have found this state to exist in the male, he has acknowledged to sexual excesses or to masturbation.

I have only stated facts and leave them to the profession, only adding that I have found the lower part of the body including genitalia and limbs of my female patients inadequately clothed.

